# The Altered States Database: Psychometric data from a systematic literature review

**DOI:** 10.1038/s41597-022-01822-4

**Published:** 2022-11-23

**Authors:** Johanna Prugger, Ekin Derdiyok, Jannis Dinkelacker, Cyril Costines, Timo T. Schmidt

**Affiliations:** 1 Psychedelic Substances Research Group, Department of Psychiatry and Neurosciences, Berlin Institute of Health, Charité Universitätsmedizin Berlin, Corporate Member of Freie Universität Berlin, Humboldt-Universität Zu Berlin, Campus Charité Mitte, Berlin, Germany; 2 International Graduate Program Medical Neurosciences, Charité Universitätsmedizin Berlin, Berlin, Germany; 3 Master Program Cognitive Neuroscience, Freie Universität Berlin, Berlin, Germany; 4 Independent researcher, Berlin, Germany; 5 Department of Psychosomatic Medicine and Psychotherapy, Medical Center - University of Freiburg, Faculty of Medicine, University of Freiburg, Freiburg, Germany; 6 Department of Education and Psychology, Freie Universität Berlin, Berlin, Germany

**Keywords:** Translational research, Psychology, Medical research

## Abstract

In this paper, we present the development of the Altered States Database (ASDB), an open-science project based on a systematic literature review. The ASDB contains psychometric questionnaire data on subjective experiences of altered states of consciousness (ASC) induced by pharmacological and non-pharmacological methods. The systematic review follows the Preferred Reporting Items for Systematic Reviews and Meta-Analyses (PRISMA) guidelines. Scientific journal articles were identified through PubMed and Web of Science. We included studies that examined ASC using the following validated questionnaires: Altered States of Consciousness Rating Scale (APZ, 5D-ASC, 11-ASC), Phenomenology of Consciousness Inventory (PCI), Hallucinogen Rating Scale (HRS), or Mystical Experience Questionnaire (MEQ30). The systematic review resulted in the inclusion of a total of 165 journal articles, whereof questionnaire data was extracted and is now available on the Open Science Framework (OSF) website (https://osf.io/8mbru) and on the ASDB website (http://alteredstatesdb.org), where questionnaire data can be easily retrieved and visualized. This data allows the calculation of comparable psychometric values of ASC experiences and of dose-response relationships of substances inducing ASC.

## Abstract (summary)

**Table:** 

Measurement(s)	Psychometric questionnaire data
Technology Type(s)	Systematic literature review (PRISMA)
Sample Characteristic - Organism	Human

## Background & Summary

In recent years there has been a growing interest in the scientific study of consciousness, including the investigation of altered states of consciousness (ASC). ASC are mental states distinct from ordinary states of consciousness and can involve profound changes in subjective experiences, such as changes in perception of the external world, of one’s own feelings, sensations, and thoughts, an altered sense of space and time, the disintegration of self-consciousness (ego dissolution), hallucinations or the experience of unity^1–5^. What varieties of experiences occur under what circumstances gained interest among clinicians, empirical scientists, philosophers, and the public^6–10^. In addition, recently, an increasing number of clinical trials started to explore the potential therapeutic effects of psychedelic drugs which can induce profound ASC experiences^11,12^.

In the experimental study of consciousness, particular attention is paid to induced ASC that are reversible and of short-term duration. These can be induced by pharmacological methods such as psychedelics, stimulants, or narcotics and non-pharmacological methods such as meditation, sensory deprivation, or breathing techniques. There is growing interest in how these experiences can be mapped and taxonomized at a phenomenological level of description, how they relate to psychiatric disorders such as schizophrenia or mania, and how they can potentially be used therapeutically^1^.

To quantitatively assess the subjective experiences of ASC, standardized and validated questionnaires for retrospective assessments have been developed^13,14^. (For more information on the questionnaires and their validation see de Deus Pontual *et al*.^15^ and Schmidt & Majić^13^). The available psychometric tools can be used to quantify different aspects of ASC phenomena, allowing for direct comparisons between induction methods, individual subjective effects, and experimental designs^1^.

The Altered States Database (ASDB, http://alteredstatesdb.org) is an open-science project that aims to provide researchers and non-scientists with easy access to valuable information concerning ASC. Here we report on the upgrade of the ASDB to be in accordance with the requirements of the Preferred Reporting Items for Systematic Reviews and Meta-Analyses (PRISMA) 2020 Statement Guidelines^16^ for reporting systematic reviews and meta-analyses and to extend the existing database by including recently published data. Articles were searched in the PubMed and Web of Science databases. After screening and identifying relevant studies, significant data were extracted and made available in the Open Science Framework (OSF, https://osf.io/8mbru)^17^. Based on a comprehensive review of currently available psychometric data on ASC, this revised version of the ASDB allows for a direct comparison of the psychological effects of different induction substances and techniques^1^. Researchers conducting clinical trials on substances or nonpharmacological methods that induce ASC can use ASDB data to compare their results with previous studies, as well as with recreational settings and related field studies. In addition, pharmacological dose-response calculations are facilitated (see Hirschfeld and Schmidt^18^).

## Methods

This article reports on the Altered States Database (ASDB; Website: http://alteredstatesdb.org), an open-science project containing extracted psychometric questionnaire data on altered states of consciousness from journal articles collected in a systematic literature review according to the PRISMA 2020 Statement Guidelines^16^.

### Eligibility criteria

This systematic review included studies on ASC experiences that used one or more standardized questionnaires to quantitatively assess the subjectively experienced qualities of ASC. A list of standardized questionnaires can be found in Table 1. Studies using both pharmacological and nonpharmacological ASC-inducing methods were included. Studies on both healthy and clinical subjects were reviewed. Only primary research data were considered.

**Table 1:** Psychometric questionnaires and their factors and scales.

Questionnaire	Versions	Scales/Factors	Original publication
Altered States of Consciousness Rating Scale	APZ	(1) Oceanic Boundlessness, (2) Dread of Ego Dissolution, (3) Visionary Restructuralization	Dittrich, 1975^19^, 1985^20^, 1998^2^
	5D-ASC	(1) Oceanic Boundlessness, (2) Dread of Ego Dissolution, (3) Visionary Restructuralization, (4) Auditory Alterations, (5) Vigilance Reduction	Bodmer *et al*.^21^ Dittrich *et al*.^22^
	11-ASC	(1) Experience of Unity, (2) Spiritual Experience, (3) Blissful State, (4) Insightfulness, (5) Disembodiment, (6) Impaired Control and Cognition, (7) Anxiety, (8) Complex Imagery, (9) Elementary Imagery, (10) Audio-Visual Synesthesia, (11) Changed Meaning of Percepts	Studerus *et al*.^3^
Phenomenology of Consciousness Inventory	PCI	(1) Positive Affect, (a.) Joy, (b.) Sexual Excitement, (c.) Love, (2) Negative Affect, (a.) Anger, (b.) Sadness, (c.) Fear, (3) Altered Experience, (a.) Altered Body Image, (b.) Altered Time Sense, (c.) Altered Perception, (d.) Altered Meaning, (4) Visual Imagery, (a.) Amount, (b.) Vividness, (5) Attention, (a.) Direction, (b.) Absorption, (6) Self Awareness, (7) Altered State of Awareness, (8) Internal Dialogue, (9) Rationality, (10) Volitional Control, (11) Memory, (12) Arousal	Pekala,1991^23^; Pekala R. J. and Levine R. L^24^.
Hallucinogen Rating Scale	HRS	(1) Somaesthesia, (2) Affect, (3) Perception, (4) Cognition, (5) Volition, (6) Intensity	Strassman *et al*.^25^
Mystical Experience Questionnaire	MEQ30	(1) Mystical, (2) Positive Mood, (3) Transcendence of time and space, (4) Ineffability	Pahnke, 1963^26^, 1966^27^; MacLean *et al*.^28^

### Information sources and article searching

An electronic search for eligible studies was conducted on the following search engines and databases: MEDLINE via PubMed and ISI Web of Science.

### Article identification using PubMed search

The PubMed search was conducted with the aim to collect journal articles which studied ASC experiences by using defined questionnaires.

The following substances that have been described to pharmacologically induce ASC were included in the PubMed search query:

*2C-B, 4-FA, 4-Fluoroamphetamine, 5-MeO-DMT, Amanita muscaria, Amphetamine, Angel dust, Atropa belladonna, Ayahuasca, Buprenorphine, Cannabidiol, CBD, Cocaine, Dimethyltryptamine, DMT, Ergine, Ergotamine, Gamma hydroxybutyric acid, GHB, Hallucinogens, Hawaiian baby woodrose, Henbane, Heroin, Hyoscyamine, Ibogaine, Kava, Ketamine, Kratom, LSA, LSD, Lysergic acid amide, Lysergic acid diethylamide, Magic mushrooms, MDA, MDMA, Methamphetamine, Morning glory, Morphine, Myristicin, Nicotine, Nitrous oxide, Nutmeg, PCP, Peyote, Phencyclidine, Pituri, Psilocybin, Psychedelics, Salvinorin, San pedro, Tetrahydrocannabinol, THC, and Triazolam.*

The following techniques reported to induce ASC non-pharmacologically were included in the PubMed search query:

*Aikido, Alternate nostril breathing, Ananda marga, Ashtanga, Autogenic training, Binaural beats, Breathwork, Capoeira, Chanting, Dancing, Dehydration, Dream machine, Drumming, Electronic gaming machine, Fasting, Flicker light, Flotation tank, Ganzfeld, Hyperventilation, Hypnosis, Hypnotic, I-OBE, Isha shoonya, Kriya, Kundalini, Kung fu, Mantra, Martial arts, Meditation, Mind machine, Nidra, Perceptual deprivation, Poker machines, Pranayama, Progressive muscle relaxation, Qigong, Repetitive speech, Runner’s high, Sahaja, Sensory deprivation, Slot machines, Stroboscopic, Sufi whirling, Sweat lodge, Tai chi, Trance, Vipassana, Yoga, Yogic breathing, and Zen training.*

The PubMed search query also included the following names of psychometric questionnaires for qualitative assessment of ASC experiences and their abbreviations:

*Abnormal Mental States Questionnaire, APZ, Altered States of Consciousness Rating Scale, 5D-ASC, Hallucinogen Rating Scale, HRS, Mystical Experience Questionnaire, MEQ, and Phenomenology of Consciousness Inventory, PCI.*

To further increase accuracy in identifying suitable journal articles and because questionnaire names or abbreviations could not be provided in the article titles or abstracts, the following “inclusion terms” were also added to the PubMed search query:

Phenomenology, Psychometric, Psychometry, Subjective effect*, Subjective experience*, and Subjectively perceived.

To exclude animal or *in-vitro* studies, as well as studies in which the above abbreviations were used in contexts other than the questionnaires or ASC-induction methods (e.g., “DMT” used for “dance movement therapy” instead of referring to the chemical substance), the PubMed search query also contained following “exclusion terms”:

*Action on salt china, Active symptom control, Alprazolam, Ambulatory surgery center, Appropriate symbol communication, Aripiprazole, Ascorbate, Ascorbic acid, Azapropazone, Cat, Cats, Dance movement therapy, Dance movement therapy, Dance/movement training, Dexmedetomidine, Disease modifying therapy, Fisher’s lsd, Gene, Genes, Hazard ratios, Hepatorenal syndrome, Least significance difference, Mice, Mouse, Percutaneous cardiovascular procedures, Percutaneous coronary intervention, Percutaneous intervention, Post hoc lsd test, Post-hoc lsd test, Primate, Primates, Rat, Rats, Rodent, Rodents, Squamous cell, Stem cell*, and *Stromal cell*.

These “exclusion terms” were chosen by manually scanning through the PubMed results of the search query without “exclusion terms” and identifying misleading uses of abbreviations etc.

The above search terms were combined with PubMed Boolean operators to detect articles describing one or more of the listed ASC-inducing methods, in addition to one or more questionnaires, or one or more “inclusion terms”, and exclude articles containing one or more “exclusion terms”. The PubMed search query also comprised the following filters:

- publication date was constrained to range from 1975 to 2021 (as the first published questionnaire contained in this review is the “Abnormal Mental States Questionnaire”, which was published in 1975),
- language filter was set to English.

To enhance the search depth, MeSH (Medical Subject Headings) terms were activated.

See Supplementary File 1 for the complete PubMed search query. The PubMed search was conducted on 2021-12-31.

### Article identification using ISI Web of Science search

To identify further studies, references of questionnaires assessing the subjective experience of ASC were tracked using the ISI Web of Science search engine. Original publications of the questionnaires of Table 1 were identified in the ISI Web of Science search engine and forward citation tracking was undertaken. Review articles were excluded. The ISI Web of Science search was conducted on 2021-12-31.

**Fig. 1:**
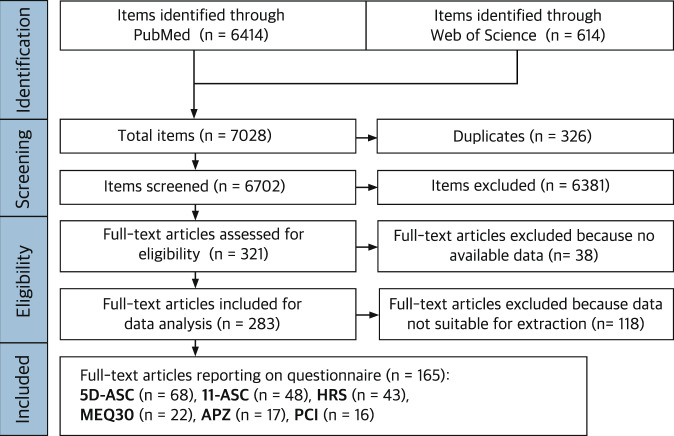
PRISMA flowchart of systematic review describing the process of study search and selection.

The process of item identification and screening is shown schematically as flow chart in Fig. 1. The PubMed search yielded 6414 items and the ISI Web of Science search yielded 614 items. The results from PubMed and ISI Web of Science were collected in the reference manager Zotero (https://www.zotero.org) and totaled 7028 items. Of these, 326 duplicates were detected and merged, leaving 6702 items eligible for the screening procedure.

### Article screening

The study selection process comprised of a first screening step in which only the title and abstract of the articles were analyzed, and a second screening step, in which full-texts and questionnaire data of the articles were examined. The aim of the first screening step was to determine whether the articles collected matched our review interest. Items were excluded if the item type was “book”, “book section” or “video recording”, if the language was not English, if they reported on animal or *in-vitro* studies, if they were reviews or other secondary literature, if they used other types of questionnaires or if the articles were not accessible. In the first screening stage, 6381 items were excluded, and 321 items were included for the full-text screening. The aim of the second screening step was to assess whether included items contain accessible and extractable data on the questionnaire results generated during the described study. For data extraction, full texts containing data on questionnaire results were collected. The second screening step resulted in the exclusion of 38 full texts that did not contain accessible data and were therefore rejected, and in the inclusion of 283 journal articles for data analysis. Thereof 118 items were excluded due to unsuitable data reporting, for example, because only total questionnaire scores or only correlation measures were reported.

### Data extraction

165 journal articles were included in the data extraction process, of which 68 contained data on the 5D-ASC, 48 on the 11-ASC, 43 on the HRS, 22 on the MEQ30, 17 on the APZ, and 16 on the PCI (note: several articles contain data from more than one questionnaire). The means and standard deviations of the responses to each of the factors and dimensions of the questionnaire described by the journal article were extracted. If only the standard error of the mean was given, it was converted to the standard deviation. When data were provided graphically only, they were extracted using *WebPlotDigitizer v4.5*.

### Results of individual studies and statistical syntheses

This systematic literature review results in the inclusion of data from 165 journal articles reporting a total of 674 datasets (experiments); these contain a total of 4689 data points as group-level summary statistics for all the factors/dimensions of all questionnaires combined (i.e., counting the data points of each dimensions/factors of a questionnaire for all datasets); these, in turn, result from a total of 17792 measurements (number of applications of all questionnaires on individual study participants). An individual dataset was defined as any unique combination of experimental conditions and questionnaire to capture that a research article may contain multiple datasets (i.e., applications of different induction methods and/or dosages result in different datasets). Table 2 reports a summary of the amount of extracted data regarding different ASC induction methods. Table 3 provides references to the articles from which data were extracted, sorted according to the different questionnaires.

**Table 2:** Summary of included data sorted according to ASC induction methods.

Induction Method		Articles	Datasets	Number of Applications	Mean sample size per dataset ± SD
Pharmacological	2C-B (4-Bromo-2,5-Dimethoxyphenethylamine)	2	2	51	25.5 ± 13.4
	4-FA (4-Fluoroamphetamine)	1	3	36	12
	5-MeO-DMT (5-Methoxy-N,N- Dimethyltryptamine)	5	15	932	62.1 ± 79.7
	DMT + MAO* Inhibitor (“Ayahuasca”)	11	20	436	21.8 ± 18.3
	DMT (N,N-Dimethyltryptamine)	9	22	295	13.4 ± 8
	DXM (Dextromethorphan)	1	7	84	12
	D-Amphetamine	1	8	32	4
	D-Methamphetamine	1	1	8	8
	Ergotamine	1	3	51	17
	Ibogaine	1	1	27	27
	Kambô (Giant Leaf Frog)	1	4	22	22
	Ketamine	24	33	599	18.1 ± 7.3
	LSD (Lysergic Acid Diethylamide)	20	66	2494	37.8 ± 151.6
	Mazindol	1	1	10	10
	MDA (3,4-Methylene dioxy amphetamine)	1	3	36	12
	MDE (3,4-Methylenedioxyethylamphetamine)	2	2	22	11 ± 4.2
	MDMA (3,4-Methylenedioxy methamphetamine, “Ecstasy”)	20	45	815	18.1 ± 9
	Mescaline (Peyote, San Pedro cacti)	1	1	12	12
	Psilocybin (“Magic Mushrooms”)	35	112	1900	17 ± 12.2
	Salvinorin-A (Salvia Divinorum)	10	69	692	8.2 ± 10
	THC (Tetrahydrocannabinol)	1	1	19	19
	Triazolam	2	4	64	16 ± 4.6
Non-pharmacological	Chanting (religious)	1	9	401	44.6 ± 27
	Drumming and Dancing	2	3	187	62.3 ± 38.9
	Flicker Light Stimulation	1	6	144	24
	Ganzfeld	3	13	306	23.5 ± 5.2
	Hetero-Hypnosis	8	20	1059	53 ± 78.7
	Kundalini Meditation	1	1	12	12
	Mind Machine	1	2	60	30
	Olfactory Epithelium Stimulus^†^	1	1	12	12
	Sweat Lodge	1	2	110	55
	Zen Meditation	1	1	14	14

This table contains, the sum of journal articles reporting on each of the identified induction method (“Articles”), the sum of experiments conducted (“Datasets”), the sum of all applications of the questionnaires on individual study participants (“Applications”), as well as the sample size per dataset given as mean ± standard deviation (“Mean sample size per dataset ± SD”).

*MAO: Monoamine oxidase ^†^Olfactory Epithelium Stimulus is not an induction method itself, but it is investigated as the mechanism underlying breathing techniques.

**Table 3:** Summary of included studies of ASC experiences.

Induction Method		APZ (3D)	5D-ASC	11-ASC	HRS	MEQ30	PCI
Pharmacological	2C-B (4-Bromo-2,5-Dimethoxyphenethylamine)				^29,30^		
	4-FA (4-Fluoroamphetamine)		^31^	^31^	^31^		
	5-MeO-DMT (5-Methoxy-N,N- Dimethyltryptamine)		^32,33^	^34^		^35–37^	
	DMT + MAO* Inhibitor (“Ayahuasca”)	^38–40^	^41^	^41^	^38,39,42–50^	^44,51,52^	
	DMT (N,N-Dimethyltryptamine)		^53,54^	^55^	^25,56–59^	^51,55^	
	DXM (Dextromethorphan)			^60^	^60,61^	^60^	
	D-Amphetamine		^62^	^62^	^42,43,63^	^62^	
	D-Methamphetamine	^64^					
	Ergotamine		^65^				
	Ibogaine		^66^				
	Kambô (Giant Leaf Frog)		^67^	^67^		^67^	^67^
	Ketamine	^68^	^53,54,69–83^	^79,84,85^	^86–89^	^85^	
	LSD (Lysergic Acid Diethylamide)		^8,62,90–95^	^62,91,92,94,96–106^		^51,62,91,92,94,105^	
	Mazindol		^107^				
	MDA (3,4-Methylene dioxy amphetamine)		^108,109^	^109^			
	MDE (3,4-Methylenedioxyethylamphetamine)	^64,110^					
	MDMA (3,4-Methylenedioxy methamphetamine, “Ecstasy”)		^62,94,111–126^	^62,94,116–119,125^	^127^	^62,94^	
	Mescaline (Peyote, San Pedro cacti)	^128^					
	Psilocybin (“Magic Mushrooms”)	^64,129–131^	^65,79,83,132–146^	^60,79,141,147–159^	^60,129–131,136,137,157^	^35,51,60,136,137,152,154,160,161^	
	Salvinorin-A (Salvia Divinorum)	^162–165^			^162–171^		
	THC (Tetrahydrocannabinol)	^172^					
	Triazolam				^61,87^		
Non-pharmacological	Chanting (religious)					^173^	
	Drumming and Dancing			^174^			^175^
	Flicker Light Stimulation		^176^	^176^			^176^
	Ganzfeld		^177,178^	^177,178^			^178,179^
	Hetero-Hypnosis						^180–187^
	Kundalini Meditation						^188^
	Mind Machine	^189^					
	Olfactory Epithelium Stimulus^†^						^190^
	Sweat Lodge	^191^					
	Zen Meditation						^192^

This table contains both pharmacological and non-pharmacological studies on ASC experiences, which were included in our systematic literature review and in the data extraction process. It categorizes studies according to which psychometric questionnaire was used to assess ASC experiences. Substances inducing ASC that do not have direct biological sources were named by the chemical formula; substances that are directly derived from biological sources were named by the active component and the species name. The street names of substances are in quotation marks. Three studies^137,141,142^ combined psilocybin administration with meditation but were included in this table only under the psilocybin category. One study^158^ investigated the effect of setting (physical and social environment during the ASC experience) on phenomenology without psilocybin being administered, yet it is included in this table under the psilocybin category because participants were led to believe that they were being administered psilocybin. We have not included substances in this table that were used as either active or passive controls in the cited studies (e.g., ketanserin, niacin, citalopram), while corresponding control-datasets are included in the ASDB.

*MAO: Monoamine oxidase. ^†^Olfactory Epithelium Stimulus is not an induction method itself, but it is investigated as the mechanism underlying breathing techniques.

In total, 145 articles report on pharmacologically induced ASC and 20 report on non-pharmacologically induced ASC. The most common questionnaire to assess pharmacologically induced ASC experiences is the 5D-ASC, (65 articles, 90 datasets, 1792 applications), followed by the 11-ASC (43 articles, 144 datasets, 2321 applications), the HRS (43 articles, 128 datasets, 1804 applications), the MEQ30 (21 articles, 58 datasets, 5183 applications), the APZ (15 articles, 45 datasets, 551 applications) and lastly the PCI (1 article, 1 dataset, 22 applications). For non-pharmacologically induced ASC experiences, the most frequently used questionnaire is the PCI (15 articles, 32 datasets, 1450 applications), followed by the 11-ASC (4 articles, 7 datasets, 151 applications), the 5D-ASC (3 articles, 6 datasets, 133 applications), the APZ (2 articles, 4 datasets, 170 applications), and lastly the MEQ30 (1 article, 9 datasets, 401 applications).

## Data Records

The results of the reported systematic literature research and the full report of extracted psychometric questionnaire data on ASC experiences are available on Open Science Framework (https://osf.io/8mbru, 10.17605/OSF.IO/8MBRU)^17^ in the folder “ASDB_v2.0_12-2021”. The psychometric questionnaire data is organized in one Microsoft Excel file per questionnaire. The data contained in the files are listed according to each individual application of the respective questionnaire. The data files are structured to fit a mySQL database structure as previously described^1^.

Following data columns are described:

- number of subjects
- control or experiment condition
- questionnaire application time
- reference of experience assessment
- subjects’ health
- questionnaire abbreviation
- PubMed ID
- DOI
- main author
- date published
- reference text
- abstract text
- paper link
- dosage quantity
- dosage unit
- info about induction
- induction method
- injection method
- pharmacological or non-pharmacological study
- laboratory or field study
- psychometric data (with as many columns needed for the various factors and dimensions, each described by mean and standard deviation, of the corresponding questionnaire)
- comments on additional information on the data extraction process such as the conversion of standard error of the mean to standard deviation.

In addition to the questionnaire data files, the OSF also contains a list of excluded studies, containing their PubMed ID, publication year, first author, and a comment on the reason for exclusion. In addition to the data availability on OSF, questionnaire data can also be retrieved and visualized on the ASDB website (http://alteredstatesdb.org), providing easy and direct access.

## Technical Validation

To validate the search strategy, a comparison was made with the 105 journal articles already included in the previous version of the ASDB (last updated 2020-12-28). The references of the articles were retrieved from OSF (https://osf.io/8mbru)^17^. The comparison showed that all articles from the older version of the ASDB were covered by the current systematic literature review. To reduce the risk of bias in study identification and selection, the study selection process was performed independently by J.P. and J.D. and subsequently cross-checked. The data extraction process was performed by J.P., E.D., and J.D. and then cross-checked as well. Any discrepancies on study eligibility and data extraction were resolved by consensus. Reasons for exclusion are documented and can be obtained together with the overall data set. No automated tools were used in screening studies, other than detecting but not merging duplicate studies in Zotero. The selection of studies should not be prone to error, as we tried to include all available studies in this research area.

## Supplementary information

Supplementary File 1

## Data Availability

No custom code was used to generate, process, or analyse the data presented in the manuscript.
