# Supplementary material for: The Altered States Database: Psychometric data from a systematic literature review
Source: Sci Data. 2022 Nov 23;9:720. doi: 10.1038/s41597-022-01822-4 (PMC9684144; doi:10.1038/s41597-022-01822-4)
Supplement: Supplementary file 1 [file 41597_2022_1822_MOESM1_ESM.pdf]

Supplementary File 1: Complete PubMed Search Query

((("hallucinogenic"[All Fields] OR "hallucinogenics"[All Fields] OR "hallucinogens"[Pharmacological Action] OR "hallucinogens"[MeSH Terms] OR "hallucinogens"[All Fields] OR "hallucinogen"[All Fields] OR ("hallucinogens"[Pharmacological Action] OR "hallucinogens"[MeSH Terms] OR "hallucinogens"[All Fields] OR "psychedelic"[All Fields] OR "psychedelics"[All Fields]) OR ("Lysergic acid diethylamide"[MeSH Terms] OR ("lysergic"[All Fields] AND "acid"[All Fields] AND "diethylamide"[All Fields]) OR "Lysergic acid diethylamide"[All Fields] OR "lsd"[All Fields]) OR "Lysergic acid diethylamide"[All Fields] OR ("ergotamine"[MeSH Terms] OR "ergotamine"[All Fields] OR "ergotamines"[MeSH Terms] OR "ergotamines"[All Fields]) OR "DMT"[All Fields] OR ("methoxydimethyltryptamines"[MeSH Terms] OR "methoxydimethyltryptamines"[All Fields] OR "5 meo dmt"[All Fields]) OR "Dimethyltryptamine"[All Fields] OR ("banisteriopsis"[MeSH Terms] OR "banisteriopsis"[All Fields] OR "ayahuasca"[All Fields]) OR ("psilocybin"[MeSH Terms] OR "psilocybin"[All Fields] OR "psilocybine"[All Fields] OR "psilocybin s"[All Fields]) OR "magic mushrooms"[All Fields] OR "amanita muscaria"[All Fields] OR ("mescaline"[MeSH Terms] OR "mescaline"[All Fields] OR "peyote"[All Fields]) OR "San Pedro"[All Fields] OR ("salvinorin a"[Supplementary Concept] OR "salvinorin a"[All Fields] OR "salvinorin"[All Fields] OR "salvinorins"[All Fields]) OR ("ibogaine"[MeSH Terms] OR "ibogaine"[All Fields] OR "ibogaine s"[All Fields]) OR "2C-B"[All Fields] OR ("n methyl 3,4 methylenedioxyamphetamine"[MeSH Terms] OR "n methyl 3 4 methylenedioxyamphetamine"[All Fields] OR "mdma"[All Fields]) OR ("amphetamine s"[All Fields] OR "amphetamines"[MeSH Terms] OR "amphetamines"[All Fields] OR "amphetaminic"[All Fields] OR "dextroamphetamine"[MeSH Terms] OR "dextroamphetamine"[All Fields] OR "amphetamine"[All Fields] OR "amphetamine"[MeSH Terms]) OR ("dextroamphetamine"[MeSH Terms] OR "dextroamphetamine"[All Fields] OR "d amphetamine"[All Fields]) OR ("methamphetamine"[MeSH Terms] OR "methamphetamine"[All Fields] OR "methamphetamine s"[All Fields] OR "methamphetamines"[All Fields]) OR "D-methamphetamine"[All Fields] OR "Methylenedioxyethylamphetamine"[All Fields] OR ("esketamine"[Supplementary Concept] OR "esketamine"[All Fields] OR "ketamine"[All Fields] OR "ketamine"[MeSH Terms] OR "ketamin"[All Fields] OR "ketamine s"[All Fields] OR "ketamines"[All Fields]) OR ("esketamine"[Supplementary Concept] OR "esketamine"[All Fields] OR "s ketamine"[All Fields]) OR ("methylphenidate"[MeSH Terms] OR "methylphenidate"[All Fields] OR "methylphenidate s"[All Fields] OR "methylphenidates"[All Fields]) OR ("modafinil"[MeSH Terms] OR "modafinil"[All Fields] OR "modafinil s"[All Fields]) OR ("naltrexone"[MeSH Terms] OR "naltrexone"[All Fields] OR "naltrexon"[All Fields] OR "naltrexone s"[All Fields]) OR ("pindolol"[MeSH Terms] OR "pindolol"[All Fields]) OR ("triazolam"[MeSH Terms] OR "triazolam"[All Fields]) OR ("niacin"[MeSH Terms] OR "niacin"[All Fields] OR "niacin s"[All Fields] OR "niacinate"[All Fields] OR "niacine"[All Fields] OR "niacins"[All Fields]) OR ("reboxetine"[MeSH Terms] OR "reboxetine"[All Fields] OR "reboxetine s"[All Fields]) OR ("haloperidol"[MeSH Terms] OR "haloperidol"[All Fields] OR "haloperidol s"[All Fields] OR "haloperidole"[All Fields]) OR ("citalopram"[MeSH Terms] OR "citalopram"[All Fields] OR "citalopram s"[All Fields]) OR ("clonidine"[MeSH Terms] OR "clonidine"[All Fields] OR "clonidin"[All Fields] OR "clonidine s"[All Fields]) OR ("aerosils"[All Fields] OR "silicon dioxide"[MeSH Terms] OR ("silicon"[All Fields] AND "dioxide"[All Fields]) OR "silicon dioxide"[All Fields] OR "aerosil"[All Fields]) OR ("mannitol"[MeSH Terms] OR "mannitol"[All Fields] OR "mannitols"[All Fields]) OR ("ketanserin"[MeSH Terms] OR "ketanserin"[All Fields] OR "ketanserine"[All Fields]) OR ("buspirone"[MeSH Terms] OR "buspirone"[All Fields] OR "buspiron"[All Fields] OR "buspirone s"[All Fields]) OR ("carvedilol"[MeSH Terms] OR "carvedilol"[All Fields] OR "carvedilol s"[All Fields]) OR ("dextromethorphan"[MeSH Terms] OR "dextromethorphan"[All Fields] OR "dextromethorphane"[All Fields]) OR ("doxazosin"[MeSH Terms] OR "doxazosin"[All Fields] OR "doxazosine"[All Fields]) OR ("duloxetin"[All Fields] OR "duloxetine hydrochloride"[MeSH Terms] OR ("duloxetine"[All Fields] AND "hydrochloride"[All Fields]) OR "duloxetine hydrochloride"[All Fields] OR "duloxetine"[All Fields] OR "duloxetine

s"[All Fields]) OR "Ganzfeld"[All Fields] OR ("hypnosis"[MeSH Terms] OR "hypnosis"[All Fields]) OR ("hypnosis"[MeSH Terms] OR "hypnosis"[All Fields] OR "hypnotism"[All Fields] OR "hypnotically"[All Fields] OR "hypnotics and sedatives"[Pharmacological Action] OR "hypnotics and sedatives"[MeSH Terms] OR ("hypnotics"[All Fields] AND "sedatives"[All Fields]) OR "hypnotics and sedatives"[All Fields] OR "hypnotic"[All Fields] OR "hypnotics"[All Fields] OR "hypnotized"[All Fields]) OR ("trance"[All Fields] OR "trances"[All Fields]) OR ("meditate"[All Fields] OR "meditated"[All Fields] OR "meditating"[All Fields] OR "meditation"[MeSH Terms] OR "meditation"[All Fields] OR "meditations"[All Fields] OR "meditational"[All Fields] OR "meditative"[All Fields] OR "meditator"[All Fields] OR "meditators"[All Fields]) OR ("mantra"[All Fields] OR "mantras"[All Fields]) OR "Breathwork"[All Fields] OR ("zen"[All Fields] AND ("education"[MeSH Subheading] OR "education"[All Fields] OR "training"[All Fields] OR "education"[MeSH Terms] OR "train"[All Fields] OR "train s"[All Fields] OR "trained"[All Fields] OR "training s"[All Fields] OR "trainings"[All Fields] OR "trains"[All Fields])) OR "Sweat lodge"[All Fields] OR "Flotation tank"[All Fields] OR "Binaural beats"[All Fields] OR "Flicker light"[All Fields] OR ("stroboscope"[All Fields] OR "stroboscopes"[All Fields] OR "stroboscopic"[All Fields] OR "stroboscopically"[All Fields]) OR "Autogenic training"[All Fields] OR "I-OBE"[All Fields]) AND ((("alter"[All Fields] OR "alterated"[All Fields] OR "alteration"[All Fields] OR "alterations"[All Fields] OR "altered"[All Fields] OR "altering"[All Fields] OR "alters"[All Fields]) AND ("state"[All Fields] OR "state s"[All Fields] OR "stated"[All Fields] OR "states"[All Fields] OR "stating"[All Fields]) AND ("consciously"[All Fields] OR "consciousness"[MeSH Terms] OR "consciousness"[All Fields] OR "consciousnesses"[All Fields]) AND ("rated"[All Fields] OR "ratee"[All Fields] OR "ratees"[All Fields] OR "rating"[All Fields] OR "ratings"[All Fields]) AND ("scale s"[All Fields] OR "scaled"[All Fields] OR "scaling"[All Fields] OR "scalings"[All Fields] OR "weights and measures"[MeSH Terms] OR ("weights"[All Fields] AND "measures"[All Fields]) OR "weights and measures"[All Fields] OR "scale"[All Fields] OR "scales"[All Fields])) OR (("alter"[All Fields] OR "alterated"[All Fields] OR "alteration"[All Fields] OR "alterations"[All Fields] OR "altered"[All Fields] OR "altering"[All Fields] OR "alters"[All Fields]) AND ("state"[All Fields] OR "state s"[All Fields] OR "stated"[All Fields] OR "states"[All Fields] OR "stating"[All Fields]) AND ("consciously"[All Fields] OR "consciousness"[MeSH Terms] OR "consciousness"[All Fields] OR "consciousnesses"[All Fields]) AND ("scale s"[All Fields] OR "scaled"[All Fields] OR "scaling"[All Fields] OR "scalings"[All Fields] OR "weights and measures"[MeSH Terms] OR ("weights"[All Fields] AND "measures"[All Fields]) OR "weights and measures"[All Fields] OR "scale"[All Fields] OR "scales"[All Fields])) OR "APZ"[All Fields] OR "ASC"[All Fields] OR "5D-ASC"[All Fields] OR (("phenomenologies"[All Fields] OR "phenomenology"[All Fields]) AND ("consciously"[All Fields] OR "consciousness"[MeSH Terms] OR "consciousness"[All Fields] OR "consciousnesses"[All Fields]) AND ("inventoried"[All Fields] OR "inventory s"[All Fields] OR "inventorying"[All Fields] OR "personality inventory"[MeSH Terms] OR ("personality"[All Fields] AND "inventory"[All Fields]) OR "personality inventory"[All Fields] OR "inventories"[All Fields] OR "equipment and supplies"[MeSH Terms] OR ("equipment"[All Fields] AND "supplies"[All Fields]) OR "equipment and supplies"[All Fields] OR "inventory"[All Fields])) OR "PCI"[All Fields] OR "Hallucinogen Rating Scale"[All Fields] OR "HRS"[All Fields] OR "Mystical Experience Questionnaire"[All Fields] OR "MEQ30"[All Fields] OR "Psychometry"[All Fields] OR ("psychometrical"[All Fields] OR "psychometrically"[All Fields] OR "psychometrics"[MeSH Terms] OR "psychometrics"[All Fields] OR "psychometric"[All Fields]) OR "subjective experience"[All Fields] OR "subjective experiences"[All Fields] OR "subjective effect"[All Fields] OR "subjective effects"[All Fields] OR "subjectively perceived"[All Fields] OR ("phenomenologies"[All Fields] OR "phenomenology"[All Fields]))) NOT ("mouse"[Title/Abstract] OR "mice"[Title/Abstract] OR "rat"[Title/Abstract] OR "rats"[Title/Abstract] OR "cat"[Title/Abstract] OR "cats"[Title/Abstract] OR "rodent"[Title/Abstract] OR "rodents"[Title/Abstract] OR "gene"[Title/Abstract] OR "genes"[Title/Abstract] OR "primate"[Title/Abstract] OR "primates"[Title/Abstract] OR "disease-modifying therapy"[Title/Abstract] OR "dance movement therapy"[Title/Abstract] OR "disease-modifying therapy"[Title/Abstract] OR "dexmedetomidine"[Title/Abstract] OR "least significance

difference"[Title/Abstract] OR "fisher's lsd"[Title/Abstract] OR "post-hoc lsd test"[Title/Abstract] OR "post-hoc lsd test"[Title/Abstract] OR "alprazolam"[Title/Abstract] OR "aripiprazole"[Title/Abstract] OR "azapropazone"[Title/Abstract] OR "squamous cell"[Title/Abstract] OR "active symptom control"[Title/Abstract] OR "stem cell"[Title/Abstract] OR "ascorbate"[Title/Abstract] OR "stromal cell"[Title/Abstract] OR "ambulatory surgery center"[Title/Abstract] OR "ascorbic acid"[Title/Abstract] OR "hazard ratios"[Title/Abstract] OR "hepatorenal syndrome"[Title/Abstract] OR "percutaneous intervention"[Title/Abstract] OR "percutaneous coronary intervention"[Title/Abstract])) AND ((journalarticle[Filter]) AND (1975:2021[pdat]))
